# Increasing Access to Kidney Transplantation for Black and Asian Patients Through Modification of the Current A2 to B Allocation Policy

**DOI:** 10.34067/KID.0000000000000297

**Published:** 2023-11-21

**Authors:** Mehdi Nayebpour, Hanaa Ibrahim, Andrew Garcia, Naoru Koizumi, Lynt B. Johnson, Clive O. Callender, J. Keith Melancon

**Affiliations:** 1 Schar School of Policy and Government, George Mason University, Fairfax, Virginia; 2 Division of Transplantation, George Washington University Hospital, Washington, DC; 3 George Washington University School of Medicine and Health Sciences, Washington, DC; 4 Department of Surgery, Howard University School of Medicine, Washington, DC

**Keywords:** health policy, kidney, organ transplant, transplant outcomes, transplantation

## Abstract

**Key Points:**

- A2 to B incompatible transplantation is not fully practiced in the country, and further policies should encourage centers to perform more blood incompatible transplants.
- Centers that currently practice A2 to B incompatible transplants should give priority to blood type B patients who are willing to accept an A organ. This will benefit Asian and Black patients.

**Background:**

The rate of A2 to B incompatible (ABO-i) kidney transplant continues to be low despite measures in the new kidney allocation system (KAS) to facilitate such transplants. This study shows how the number of ABO-i transplants could increase if KAS policies were used to their fullest extent through a boost in ABO-i priority points.

**Method:**

Transplant outcomes were predicted using the Kidney Pancreas Simulated Allocation Model, preloaded with national data of 2010. We used this simulation to compare KAS with a new intervention in which priority equal to cPRA=100 has been given to blood type B candidates who are willing to accept an A blood type organ.

**Results:**

The number of Black recipients increased by 375 (from 35% of the total recipient population to 38.7%), the number of blood type B Blacks increased by 65 (from 8% of the total recipient population to 9%), and the number of blood type B Black patients receiving blood type A kidneys increased by 49 (from 2% of the total recipient population to 2.5%). The same change occurred for Asians, particularly blood type B Asians (from 0.54% of the total recipient population to 0.7%). The average wait time notably decreased by 27 days for blood type B Black patients. In the proposed scenario, 263 blood type B Black patients received a blood type A organ (2.5% of the total recipient population) while only 181 (1.1%) of such transplants were performed in 2021. These results signify a considerable opportunity loss of ABO-i transplants for Black patients.

**Conclusions:**

If this policy was universally adopted, we would expect to see an overall increase in A2 to B transplantation, but in reality, not all centers perform ABO-i transplantation. Thus, adopting this policy would incentivize other centers to perform more subtyping of A-type kidneys, and it would increase access to organs for blood type B Asian and Black patients in centers where ABO-i transplantation already takes place.

## Visual Abstract

**Figure:**
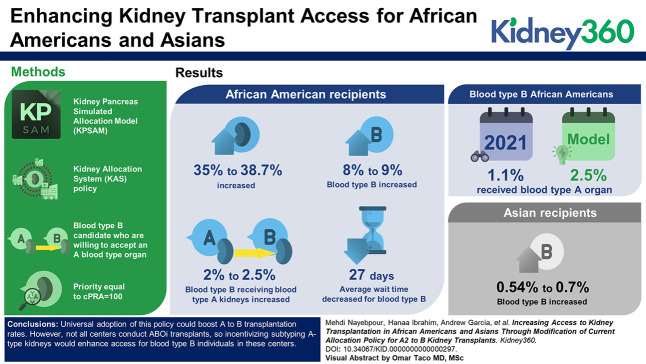

## Introduction

In the decades following the first successful kidney transplantation in the United States in 1954, there has been steady progress in achieving equity for this life-saving intervention. Changes to kidney allocation policy within the past decade, particularly for deceased donor kidney transplantation (DDKT), sought to mitigate disparity in access to transplantation among Black patients.^1^ One early obstacle in achieving maximum utilization of eligible organs has been incompatibility of ABO blood groups between donors and recipients. Transplanting a kidney from an ABO-incompatible donor was once believed to lead to graft rejection and failure.^2^ Years of research and experience spanning as far back as the 1980s demonstrated that kidney transplantation from donors with the A2 or A2B subtypes to blood type B recipients is possible when anti-A2 IgG titers were low in recipients.^2^ Such transplant patients achieved similar long-term outcomes to ABO-compatible kidney allografts.^2–4^ Prior research estimates that the frequency of the A2 phenotype among A-type individuals in the general population is close to 20%, indicating that a significant number of the population has this phenotype.^5^ In addition, graft survival in B-type recipients matched by this fashion remains similar to compatible blood type organs, indicating a vital opportunity of transplantation for individuals with a B phenotype.^6,7^ These findings were crucial to the eventual implementation of the kidney allocation system (KAS) by the Organ Procurement and Transplantation Network (OPTN) as an intervention to decrease disparity in transplantation. While this intervention increased the net number of A2/A2B to B transplants, disparity still persists among Black and Asian patients. This study highlights the reasons behind this disparity and provides recommendations to increase ABO-i transplants among Black and Asian patients on the basis of the results of a simulation. This simulation reflects the effect of increasing the priority points of ABO-i transplants in the KAS policy. We evaluate the possible consequences of ABO-i transplants if all transplant centers universally adopted and performed ABO-i transplantation with a novel priority allocation.

### Implementation of the KAS to Decrease Disparity

Black patients are the second highest population with blood type B in the United States after Asian patients (18% versus 25%, respectively).^8^ They are also the most common group in the United States to develop ESKD, often at an incidence rate more than three times higher than their White counterparts.^9^ The reasons for this are complex and multifaceted, including factors such as genetic predisposition, socioeconomic status, or other social determinants of health.^10,11^ Studies have shown that Black patients historically had diminished access to kidney transplantation, both for living and deceased donation, compared with non-Hispanic White patients.^12^ By 2013, and before implementation of the KAS, blood group B candidates, many of whom were Black patients, represented 16% of the waiting list, but only 13% of transplant recipients.^13^

The KAS, implemented on December 4, 2014, was designed to decrease disparities in transplantation by implementing new changes to the allocation algorithm, including allocating blood subgroup A2/A2B deceased-donor kidneys to candidates with blood type B.^14^ This change was made on the basis of improved long-term survival outcomes when the OPTN implemented variance of practice policies in 2002 to allow DDKT with A2 donors to B recipients. This prospective variance study showed an increase in access to kidney transplantation for minority candidates by as much as 36%, depending on donor service area in the study.^15^

A major contingency of the KAS policy is identifying the kidneys with the A2 subtype by requiring subtyping for donors with primary blood type A and optional for those with blood type AB. The official policy dictates that if there are conflicting or indeterminate subtype results, the subtype does not need to be reported to the OPTN, and the deceased donor must be allocated on the basis of the primary blood type.^16^ Organ Procurement Organizations (OPOs) are responsible for creating their own protocols to address conflicting results, including documentation of reasons why subtype tests could not be completed.^16^

### Persistence of Disparity After KAS Implementation

In the years following the implementation of KAS, reports have shown an increased access to kidneys in minority populations, yet the gap between Black patients and their counterparts still persists. A 2018 study by Martins *et al.* involving adult blood type B DDKT recipients from 2013 to 2017 examined trends in transplantation before and after the implementation of the new KAS policy. Despite finding a 4.9-fold increase in the likelihood of transplanting A2 to B DDKT in the post-implementation period, there was no evidence of improvement in receiving A2 to B DDKT among Black patients compared with non-Hispanic White patients.^12^ A 2021 study by Stern *et al.* compared rates of A2/A2B to B recipients between 2009–2014 and 2014–2019 after implementation of the KAS. They found an increase from 17 to 76 in the number of total transplant centers performing such transplants. However, 157 transplant centers did not perform a single A2/A2B to B transplant at this time.^14^ More importantly, they found that from 2015 to 2019, only 56.4% of A/AB donors were subtyped after implementation of the KAS.^14^ The authors also found that 1161 A/AB donors went unrecognized as possible A2/A2B donors because of lack of subtyping. With the assumption that each candidate donated both kidneys, 2322 possible kidneys went unrecognized as potentially compatible with B group patients, undermining the allocation policies of the KAS. Even half of this number would have had a meaningful effect, leading to a potential 24% increase in the rate of B-recipient DDKT in the post-KAS period.^14^ The reason behind the lack of subtyping is multifactorial. Frequently cited issues, however, include donor transfusions or discordant testing.^5,17^ A major limitation in realizing the reason behind lack of subtyping is that centers do not record nor provide explanations of why subtyping was not performed. Having recorded explanations may be one avenue for possible modifications and improvements in the KAS policy to maximize the potential benefits of ABO-i transplantation.^14^

## Methods

We performed a simulation to measure the effect of universal implementation of A2 to B kidney transplantation on Black and Asian patients by using the Kidney-Pancreas Simulated Allocation Model (KPSAM—version 2015), a simulation software developed by the US Scientific Registry of Transplant Recipients (SRTR). The software is preloaded with the actual 2010 transplant waiting list, organ, and patient arrivals. We were not able to use a more recent patient cohort because of the limitations of the KPSAM software that can only use the 2010 data. This program is specifically designed to support studies which compare alternative organ allocation policies by changing the allocation rules and analyzing the outcomes. The allocation process of KPSAM contains a few random elements (*e.g.*, probability of acceptance decision, life expectancy). To account for such random variables, the program runs for 10 times with the same set of allocation rules and waitlist and organs and finally determines the average outcome. This range reflects variability of the simulation modeling, not variability in actual organ allocations. In this study, three simulation scenarios were developed and the outcomes were compared: (*1*) the pre-KAS model to reflect the kidney allocation policy before December 4, 2014; (*2*) the current KAS policy; and (*3*) the *A_to_B_Boosted* scenario, which is the same as KAS while giving extra kidney priority points to blood type B patients who are willing to accept an A/AB organ (the extra points are given to such candidates equal to patients with calculated Panel Reactive Antibody (cPRA)=100). Based on the documentation of KPSAM, the software has the ability to consider titer levels for A2 to B transplants, allowing for low anti–A-titer recipients to receive a transplant. The KPSAM software documentation does not specify the cutoff point for anti-A titer. While a general consensus asserts an anti-A2 IgG threshold of ≤1:8 as the eligibility for an A2 to B transplant, the specific titer cutoffs vary by center.^2,15^ In our transplant program located in an urban setting, for instance, recipient A2 or A antibody IgG titers <1:16 are considered low enough.

Regarding statistical analysis, it is critical to note that because KPSAM uses the same donors and candidates from the calendar year 2010 in each simulation run, they are not independent samples; therefore, statistical tests of comparisons are not possible. Instead, we present the average of ten simulation runs. This is common practice in studies that use KPSAM.^18^ The number of transplants in each scenario was compared, distinguishing by blood type and race. In addition, we compared total wait time, discarded organs, and waitlist death in each scenario. The total number of A2 to B transplants in 2021 is also presented as an index of how many ABO-i transplant surgeries currently occur in the country. Notably, KPSAM uses four categories for race: White, Black, Hispanic, and all others. KPSAM does not specify Asians, but with some level of certainty, we are assuming that the “all others” category is relatively a proper representation for Asians because on the basis of the OPTN/SRTR 2010 Annual Data Report, the actual proportion of Asians on the waitlist at the beginning of 2010 was 7.6% and of unknown races was 1.3%,^24^ while in KPSAM, the proportion of all others is 8.7%. This shows that in the “all others” category, approximately 85% are Asians.

## Results

**Figure 1:**
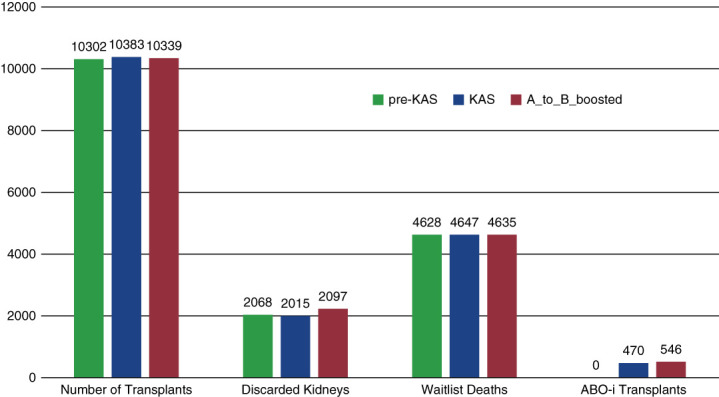
Number of transplants, discarded kidneys, deaths on waitlist, and ABO-i transplants.

Figure 1 shows the total number of transplants, discarded kidneys, and deaths on the waitlist and the number of ABO-i transplants in each scenario. *KAS* has the highest number of transplants (10,383) and the lowest number of discarded kidneys (2,015). The *A_to_B_Boosted* scenario resulted in a slightly lower number of transplants (10,339), which is 0.42% less than *KAS*, and a higher number of discarded kidneys (2,097), but a lower number of waitlist deaths compared to *KAS* (4635 versus 4647, respectively). As expected, the *A_to_B_Boosted* scenario had the highest number of ABO-i transplants (546, 5.25% of total transplants) compared to KAS (470, 4.54% of total transplants).

Tables 1 and 2 summarize the number of kidney transplants and ABO-i transplants in *KAS* and *A_to_B_Boosted* by race. The number of Black recipients noticeably increased from 3630 in KAS to 4005 in the *A_to_B_Boosted* scenario (from 35% of the total transplant population to 38.7%, respectively). The number of Asian recipients rose from 736 in KAS to 758 in the *A_to_B_Boosted* scenario (from 7.1% of the total transplant population to 7.3%, respectively). This resulted in a noticeable decrease in the number of White recipients, from 4512 in KAS to 4079 in the *A_to_B_Boosted* scenario (from 43.5% of the total transplant population to 39%, respectively). The number of Hispanic recipients did not change. The same pattern was noted among the ABO-i transplants, that is, the number and proportion of Black and Asian recipients noticeably increased, from 214 (45.6%) to 263 (48.2%) and from 57 (12.1%) to 72 (13.2), respectively. In general, the number of ABO-i transplants increased from 470 in the KAS scenario to 546 in the *A_to_B_Boosted* scenario (from 4.52% of the total recipient population to 5.28%, respectively). In reality, however, only 412 ABO-i transplants occurred in 2021 (2.59% of the total recipient population).

**Table 1:** Number of kidney transplants in the KAS and *A_to_B_Boosted* scenarios by recipient race

Race	KAS, *n* (%)	*A_to_B_Boosted*, *n* (%)
Black	3630 (35)	4005 (38.7)
White	4512 (43.5)	4079 (39)
Hispanic	1503 (14.4)	1497 (14.4)
Asian	736 (7.1)	758 (7.3)
Total	10,383	10,339

KAS, kidney allocation system.

**Table 2:** Total number of ABO-i kidney transplants in the KAS and *A_to_B_Boosted* scenarios by recipient race

Race	KAS, *n* (%)	*A_to_B_Boosted*, *n* (%)
Black	214 (45.6)	263 (48.24)
White	152 (32.4)	158 (29.03)
Hispanic	46 (9.8)	53 (9.51)
Asian	57 (12.1)	72 (13.22)
Total	470	546

KAS, kidney allocation system.

**Figure 2:**
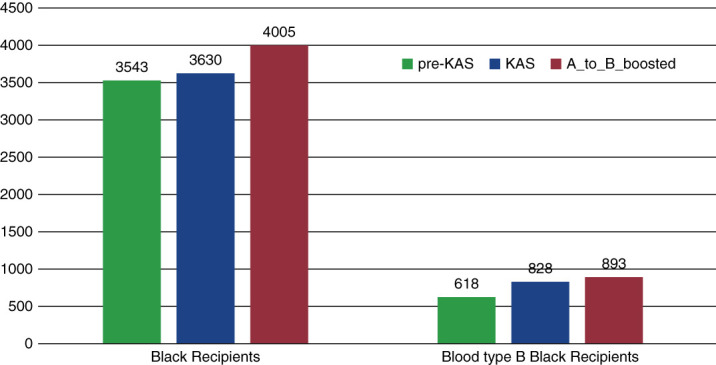
Black and blood type B Black recipients across all scenarios.

Figure 2 and Table 3 present the breakdown of the recipients in the *KAS* and *A_to_B_Boosted* scenarios by recipient blood type and race. In the *A_to_B_Boosted* scenario, the number of Black recipients of all blood types increased compared to KAS; particularly, the number of blood type B Black recipients rose from 828 in KAS to 893 in the *A_to_B_Boosted* scenario. Among Asians, the number of recipients of all blood types remained relatively the same, except those with blood type B, which increased from 209 in KAS to 226 in the *A_to_B_Boosted* scenario.

**Table 3:** Breakdown of the number of kidney transplants in the KAS and *A_to_B_Boosted* scenarios by recipient blood type and race

Race	A		AB		B		O	
	KAS	*A_to_B_Boosted*	KAS	*A_to_B_Boosted*	KAS	*A_to_B_Boosted*	KAS	*A_to_B_Boosted*
Black	852	938	218	226	828	893	1731	1947
White	1762	1620	250	224	573	566	1926	1668
Hispanic	433	437	52	48	174	171	841	839
Asian	176	182	67	64	209	226	282	285
Total	3225	3178	589	563	1787	1858	4782	4740

KAS, kidney allocation system.

**Figure 3:**
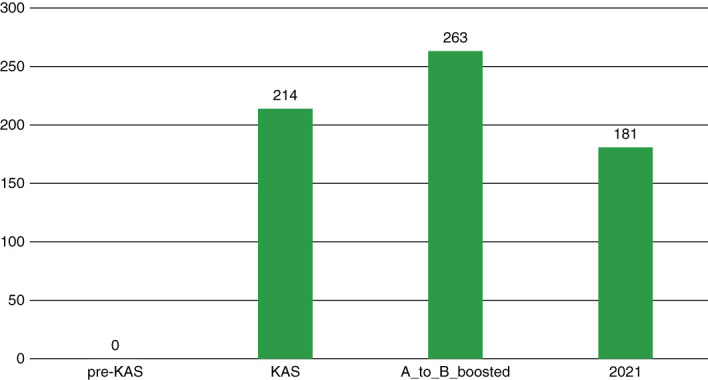
The number of A2 to B transplants for blood type B Black recipients.

Furthermore, by focusing on Black and Asian recipients with blood type B, we noticed that the number of A2 to B transplants rose from 214 in KAS to 263 in the *A_to_B_Boosted* scenario for Black patients (from 2% of the total transplant population to 2.5%, respectively) and from 57 to 72 for Asians (from 0.54% of the total transplant population to 0.7%, respectively). Figure 3 illustrates these results. In the *pre-KAS* scenario, as expected, there are no such cases. The reality, however, is that in 2021, there were only 181 A2 organs transplanted to blood type B Black recipients (1.14% of the total transplant population) and only 68 A2 organs transplanted to blood type B Asian recipients (0.42% of the total transplant population). The number of ABO-i transplants in 2021 is presented only to provide a scale of how many of such transplants occur in reality. We cannot perform statistical analysis to compare the outcomes of our simulation and the actual outcomes of 2021 because they are from two different cohorts.

**Figure 4:**
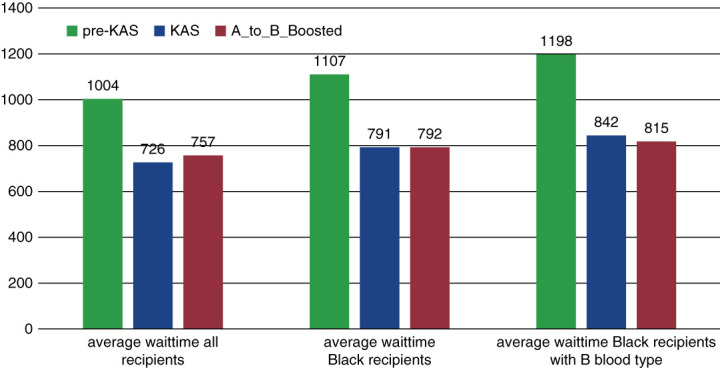
Average wait time for all recipients, Black recipients, and blood type B Black recipients.

Figure 4 shows the average wait time for all recipients, Black recipients, and blood type B Black recipients. The *pre-KAS* scenario had the longest wait time compared to all other scenarios. The average wait time for Black recipients was the same for both *KAS* and *A_to_B_Boosted* scenarios (791 versus 792 days, respectively), but the average wait time for blood type B Black recipients was notably shorter in the *A_to_B_Boosted* scenario compared to *KAS* (815 versus 842 days, respectively).

Table 4 is presented to compare the demographic and clinical characteristics of the 2010 and 2021 waitlists.^19^ Chi-squared test was performed to test whether the composition of race, blood type, time on waitlist, and age were different among the two waitlist populations. There was no statistically significant difference. It is noteworthy that 2021 is the latest available data for kidney transplant outcomes, and it is relatively distant enough from the effects of coronavirus disease 2019.

**Table 4:** Demographic and clinical characteristics comparison of the 2010 and 2021 waitlists

%	2010	2021	*P* Value
Black	34.9	32.0	Chi-squared test *P* = 0.88
Hispanic	18	20.6	
Asian	7.6	10.0	
White	38.1	35.8	
Male	59	61.9	*P* < 0.001
Previous transplant	15.9	11.6	*P* < 0.001
A blood type	28.6	26.8	Chi-squared test *P* = 0.98
B blood type	16.2	16.6	
AB blood type	3	2.5	
O blood type	52.2	54.1	
cPRA <1%	64.0	60.12	Chi-squared test *P* = 0.82
1≤cPRA<20	6.1	8.91	
20≤cPRA<80	14	16.29	
80≤cPRA≤100	15.9	14.68	
Wait time <1 yr	32.3	32.9	Chi-squared test *P* = 0.98
Wait time 1–2 yr	23.0	20	
Wait time 2–3 yr	16.7	16.6	
Wait time 3–5 yr	17.1	17.7	
Wait time>5 yr	11.0	12.9	
Diabetes	30.4	38.7	*P* < 0.001
Age 18–34 yr	10.1	7.8	Chi-squared test *P* = 0.60
Age 35–49 yr	28.2	23.3	
Age 50–64 yr	43.4	43.4	
Age>65 yr	18.3	25.4	

cPRA, calculated Panel Reactive Antibody.

## Discussion

The actual outcomes of *KAS* in 2021 and the simulated *A_to_B_Boosted* model regarding the number of A2 to B transplants in general and Black and Asian patients in particular demonstrate the missed opportunity for potential transplantation if all transplant centers practiced high-priority A2 to B transplantation. In 2021, there were only 412 ABO-i transplants (2.59% of the total recipient population) while the simulated KAS and the *A_to_B_Boosted* model produced 470 and 546 ABO-i transplants (4.52% and 5.25% of the total recipient population), respectively. Furthermore, in 2021, there were 181 A2/A2B kidneys transplanted to blood type B Black patients (1.1% of the total transplants), compared to 263 in the simulated *A_to_B_Boosted* model (2.5% of the total transplants). On the basis of these outcomes, if all transplant centers practiced A2 to B transplantation and considered boosting the kidney points of recipients who are willing to accept such transplants by the points equal to cPRA=100, the proportion of such recipients would rise from 1.1% to 2.5%, which is equal to 215 extra blood type A kidneys transplanted to blood type B Black patients. Our simulation results suggest that implementing *KAS* with added priority for A2 to B transplantation leads to a higher rate of transplantation for Black patients, shorter wait time, and lower rate of waitlist deaths for them. In addition, this scenario favors blood type B Asians, who are known to have longer median waiting time than either Black and White patients.^22^ Asians also possess the largest portion of blood type B in the US population,^23^ thus making this scenario particularly advantageous for them.

The simulated model in KPSAM does not account for the challenges and obstacles that transplant centers must face to perform A2/A2B to B transplantation. The simulation assumes that all centers can perform such transplants. Granted that this is not the case, the KAS policy is not being implemented to its full potential, and blood type B Black and Asian patients are still the least served candidates in the country. Similar studies that have surveyed A2 to B transplants in the country have reached the same conclusion.^6^ The results of the simulated KAS scenario show what would be the outcome if all centers practiced ABO-i transplantation. However, not all centers practice ABO-i transplantation. That is why the *A_to_B_Boosted* scenario takes a step forward to further encourage ABO-i transplantation.

The results of our simulated scenarios show if blood type B candidates who are willing to accept an A kidney receive extra kidney points equal to cPRA=100, the outcome will benefit Black and Asian patients. We particularly looked at the effect of our proposed scenario on highly sensitized patients with cPRA=100 because they are the hardest population to match. In the simulated KAS, there were 2.04% recipients (2121) with cPRA equal to 100 and in the *B_to_A_Boosted* scenario, there were 2.03% recipients (2103) with cPRA equal to 100, which shows little to no effect on this highly sensitized population. Greater priority and uniform participation in ABO-i transplants under the current KAS policy would help A2 to B transplantation reach its potential level. Shaffer *et al.* reported that, in 2017, only 18% of transplant centers practiced A2 to B transplantation.^6^ Other centers did not have the capacity to cover the costs or to perform necessary steps, such as titer measurement or subtyping. Owing to the small size of many transplant centers, they may never have the financial incentive to perform A2 to B transplantation. Thus, to remedy these shortcomings, we support the implementation of the A_to_B_Boosted policy in centers where ABO-i transplantation is already being practiced. This policy will let such centers to perform more ABO-i transplants than before.

The reasons for underutilization of the policies enacted in the KAS are multifaceted. Previous reports have cited concern over pretransplant titers, patient eligibility, and increased costs as major barriers to implementation.^5,17^ A 2019 retrospective cohort analysis by Shafer *et al.* sought to clarify the costs and implications of post-transplant anti-A titers on outcomes of A2 to B transplants. In a sample of 29 A2 to B transplants and 50 B to B transplants, they found that A2 to B transplants were associated with approximately $23,000 more in total hospital costs and approximately $21,400 more in net organ acquisition costs, compared with B to B transplants.^6^ Significantly, there were also pretransplant laboratory costs alone amounting to $76,550, excluding additional administrative and coordinator time costs. The authors attributed a large increase in the post-transplant costs to increased dialysis and pharmacy costs associated with their protocol for high IgG/IgM titer recipients. However, they found that anti-A titers in the post-transplant period did not correlate with 1- or 2-year post-transplant outcomes and thus seem to be unnecessary to monitor routinely. It is worth mentioning that the frequency of measurement of pretransplant anti-A titers is up to individual centers per United Network for Organ Sharing (UNOS) policy, without clear data or guidelines in the setting of intrapatient and interlaboratory variability in titer measurements.^6^

Risk aversion is another reason behind the lack of ABO-i transplants. All transplant programs are evaluated by UNOS/OPTN and Membership and Professional Standards and the Centers for Medicare & Medicaid Services with biannual reports of their outcomes. Poor performing programs are sanctioned, and therefore, all transplant programs must consistently strive to maintain stellar transplant outcomes. Treating patients with ESKD by default is challenging because of various comorbid conditions including diabetes mellitus, cardiovascular disease, and peripheral vascular disease, among others. These conditions tend to make kidney transplant programs risk-averse, and therefore, they seek to transplant patients with the best likelihood of outcome. ABO-i transplantation is perceived to be risky while evidence proves otherwise. By implementing the suggested policy to boost A2 to B matches in centers where ABO-i transplantation is already practiced, we envision that other centers might follow suit and start to practice it after witnessing the benefits of ABO-i transplantation. The same path occurred in the practice of desensitization and kidney paired donation (KPD). Desensitization protocols initially emerged in 1990s, and throughout the following decades, they were refined until they became a part of the KAS allocation policy and were widely practiced in transplant centers.^20^ In the case of KPD, the first one was performed in 2000. Initially, KPD programs were small and ran in only a few individual transplant centers, but now, several centers and multicenter KPD clearinghouses operate in the country.^21^ The limitations of this study have been described throughout the article and particularly in the methods section, but it is important to reiterate them. KPSAM is only able to use the 2010 cohort in its simulation. Although the demographic characteristics of the waitlist are not different from the 2021 waitlist, it prevents the opportunity to compare the simulation outcome with the real outcomes of 2021. In addition, the number of patients who are willing to accept an incompatible organ may differ in the 2010 and 2021 populations, but we do not have sufficient data to analyze that. This is not a major problem because the focus of this study was to compare the different simulation scenarios using only the 2010 patient population. The other limitation of our results lies in the fact that KPSAM assumes that all centers can and will perform ABO-i transplant, whereas, in reality, only a fraction of them has the capacity to do so. We acknowledged this limitation and proposed the idea of enacting this policy only in centers where ABO-i transplantation already occurs. Our results can be an image of the future for policymakers in which all centers perform ABO-i transplantation. As mentioned in the methods section, we are not able to perform statistical significance tests on the simulation results because KPSAM uses the same set of input data and runs it for ten times; hence, we can only present and compare the average of the ten runs.

## Recommendations

Access to organs for Black and Asian and Asian patients continues to be significantly less compared with their counterparts. Although the new KAS policy resulted in some success to decrease disparity for Black patients by allowing ABO-i transplantation, evidence shows that this practice has not reached its full potential. On the basis of the results of our simulation, we support the following measures to increase the number of ABO-i transplants in Black and Asian patients: (*1*) increasing the priority points equal to cPRA=100 for recipients who are willing to accept an ABO-i transplant in centers that already practice ABO-i transplantation and (*2*) modifying the KAS policy to require a rationale and explanation for not subtyping at the center and OPO levels. By implementing these two changes, UNOS, OPOs, and transplant centers would be taking a major step in decreasing disparity in transplantation for historically underserved minority groups, such as Black and Asian patients. These suggestions need to be validated and examined in a limited number of centers. Furthermore, the simulation could be updated by using the most recent waitlist, patient, and organ arrival data. This requires coordination with SRTR and reprogramming the software.

## Data Availability

Previously published data were used for this study. KPSAM—version 2015, a simulation software developed by the US Scientific Registry of Transplant Recipients (SRTR).
